# Controversies

**Published:** 1896-11

**Authors:** 


### CONTROVERSIES.

We do not believe that medical journal or newspaper controversies do the participants any good, but that they are rather injurious to both sides. We are sorry one has arisen here, but we are pleased to be able to learn some valuable truths from it. Outsiders get a great deal of otherwise unobtainable fact from such disputes, and parties indirectly interested are often shown the opinion which others hold of their position. So while the present dispute will do those immediately interested no good, we expect it to have a salutary effect in other directions. Some excellent writing is being done meanwhile and we are enjoying it greatly. H.

